# Temporoparietal Headache as the Initial Presenting Symptom of a Massive Aortic Dissection

**DOI:** 10.1155/2015/626825

**Published:** 2015-05-06

**Authors:** Manan Parikh, Abhinav Agrawal, Braghadheeswar Thyagarajan, Sayee Sundar Alagusundaramoorthy, James Martin

**Affiliations:** ^1^Department of Internal Medicine, Monmouth Medical Center, Long Branch, NJ 07740, USA; ^2^Department of Emergency Medicine, Monmouth Medical Center, Long Branch, NJ 07740, USA

## Abstract

Aortic dissection is a life-threatening medical emergency often presenting with severe chest pain and acute hemodynamic compromise. The presentation of aortic dissection can sometimes be different thus leading to a challenge in prompt diagnosis and treatment as demonstrated by the following presentation and discussion. We present a case of a 71-year-old male who presented to the emergency department with complaints of left sided temporoparietal headache and was eventually diagnosed with a thoracic aortic dissection involving the ascending aorta and descending aorta, with an intramural hematoma in the descending aorta. This case illustrates the importance of keeping in mind aortic dissection as a differential diagnosis in patients with acute onset headaches in which any intracranial source of headache is not found.

## 1. Introduction

Aortic dissection is a relatively uncommon, though catastrophic, illness often presenting with severe chest pain and acute hemodynamic compromise. The presentation of aortic dissection can sometimes be different thus leading to a challenge in prompt diagnosis and treatment as demonstrated by the following presentation and discussion.

## 2. Case Presentation

This is a case of 71-year-old Caucasian male with past medical history significant for choroideremia who presented to the emergency department (ED) with complaints of left sided temporoparietal headache upon waking up on the day of admission.

Patient in his thirties had progressive vision loss and was diagnosed as choroideremia with the deletion of REP 1. He became completely blind by the age of 50. The other significant past medical history includes ablation for supraventricular tachycardia around 20 years ago, coronary artery disease with percutaneous coronary intervention in the left anterior descending artery around 10 years ago, hypertension, and hyperlipidemia. His social history is significant for no history of smoking, alcohol consumption, and drug abuse. His family history is significant for choroideremia in his brother (complete blindness) and carrier state in his sister (asymptomatic). His medications included aspirin, clopidogrel, losartan, and pravastatin.

**Figure 1:**
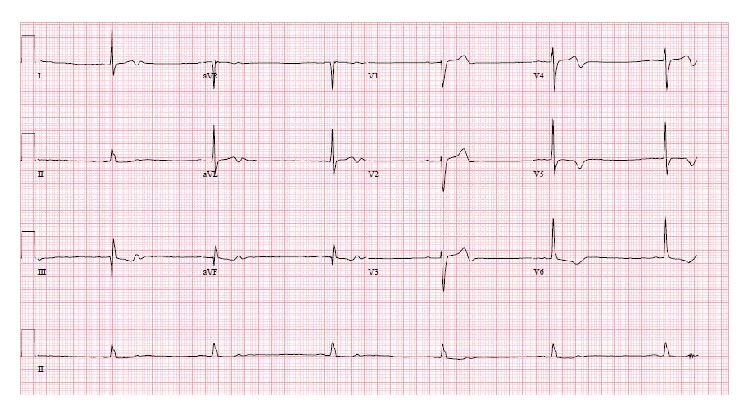
EKG showing sinus bradycardia with nonspecific ST-T wave abnormalities.

**Figure 2:**
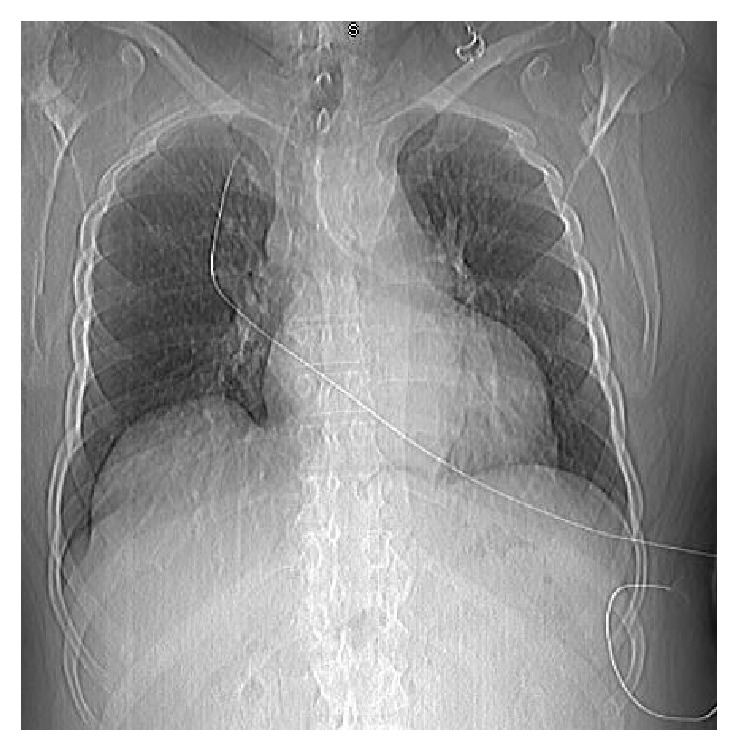
Chest X-ray showing prominence of thoracic aorta, no acute pulmonary disease.

On the day of admission, the patient woke up with a severe left sided temporoparietal headache with no numbness or weakness. Concerning this symptom, the patient contacted his primary care physician who advised the patient to go to the emergency department. His initial vitals in the ED were significant for blood pressure of 107/49 mm of hg and a heart rate of 38 beats per minute. The lab work was unremarkable with negative troponins; an ECG (Figure 1) that was done was significant for sinus bradycardia with some T wave abnormalities in lead V3 to V6 and the initial chest X-ray (Figure 2) was significant for prominence of the thoracic aorta. His physical examination was unremarkable except for his vision where the patient was able to perceive only shadows and otherwise he was completely blind. He had no focal neurological deficits. The differential at this time due to his symptoms and physical exam was possibly a pulmonary embolism versus any intracranial hemorrhage versus acute coronary syndrome. The repeat blood pressure bilaterally showed 134/61 mm of hg on the right arm and 123/53 mm of hg on the left arm with a heart rate of around 49 beats per minute.

**Figure 3:**
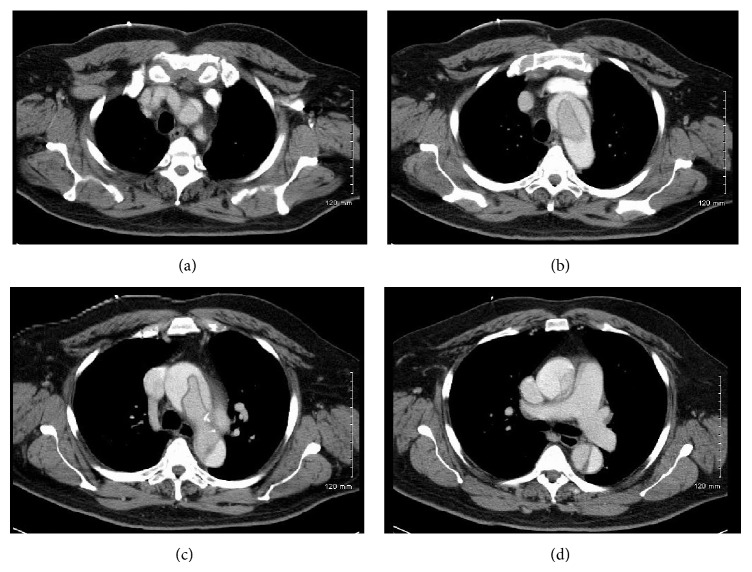
CT scan with contrast showing a thoracic aortic dissection involving the ascending aorta and descending aorta, with an intramural hematoma in the descending aorta. (a)–(d) show coronal sections at different levels.

Concerning the persistent nature of the headache and prominent thoracic aorta on the X-ray, a CT head without contrast was done which was unremarkable and a CT of the chest with contrast (Figures 3(a)–3(d)) was also done which was significant for a thoracic aortic dissection involving the ascending aorta and descending aorta, with an intramural hematoma in the descending aorta. The dissection extended superiorly to the brachiocephalic/left common carotid trunk. Concerning the life-threatening emergency of the situation it was decided that the patient needed surgical intervention. His vitals continued to be unchanged with no worsening of symptoms. Patient was emergently transferred to a tertiary care center. The patient underwent an ascending aortic replacement and hemiarch replacement as well as a suspension of the aortic valve. Patient was subsequently discharged with a stable follow-up course.

## 3. Discussion

Aortic dissection is an uncommon medical emergency with incidence of around 3 per 100,000 person-years [1]. Despite the current advancements in cardiothoracic surgery it remains a lethal event with extremely variable case fatality rate. Surgical mortality rate has been noted to be ranging from 15 to 30% for acute aortic dissection in some reviews [2]. Dissection is a dynamic process that can rapidly evolve and progress to hemodynamic compromise; therefore urgency in diagnosis remains essential. Over 90% of cases classically present with one or more of the following findings: (1) abrupt onset sharp chest or abdominal pain, (2) chest radiograph suggestive of aortic or mediastinal widening, and (3) a variation in pulse or blood pressure [3]. Despite the chest pain being the most common complaint, diagnosis of aortic dissection is often missed in absence of any type of pain in about 6% cases [4]. Common extra cardiac manifestations are neurological deficits in about 18–30% cases as well as syncope in about 13% but rare cases of dissection presenting as gastrointestinal hemorrhage, pleural effusion, intestinal ischemia, or fever of unknown origin have also been previously described [5–10]. Of the neurological manifestations stroke, spinal cord ischemia, and hypoxic encephalopathy as well as ischemic neuropathy are commonly described. But only isolated cases of vertigo, uniocular blindness, or headache have been described as the presenting manifestation of aortic dissection [11].

On extensive review of medical literature we only found a handful of cases of aortic dissection presenting as headache. A literature search using the key words “aortic dissection” and “headache” was performed using Ovid, MEDLINE, and PubMed. Singh et al. [12] have reported a case of patient presenting bifrontal headache that subsequently became hemodynamically unstable and was found to have Stanford type A aortic dissection. Ko and Park [13] have reported a case where a patient that presented with bifrontal headache on further evaluation was found to have common carotid artery dissection along with aortic dissection. Our case demonstrates a similar but unique scenario where patient presented with frontal headache and remained hemodynamically stable prior to his diagnosis of the condition and was subsequently found to have an aortic dissection. Headaches can be seen in patients with primary carotid artery dissection [14]. Headache in these situations can be due to distension of the carotid artery, thus in turn stimulating pain receptors [15]. It has also been suggested that ischemia due to reduced blood flow resulting from an aortic dissection may stimulate the depolarizing sensory fibers in the pericarotid cavernous sinus plexus and lead to headache [12]. The aortic dissection extended to the left brachiocephalic trunk along with the left common carotid trunk, which might explain the cause of left sided temporoparietal headache in this patient.

Upon suspicion, the diagnosis of aortic dissection is made with CT scan with angiography or echocardiography or via magnetic resonance imaging. Based on the imaging studies patients are further classified. The DeBakey classification has three types: type I dissections originate in the ascending aorta and extend to at least the aortic arch; type II dissections involve the ascending aorta only; and type III dissections begin in the descending aorta, usually just distal to the left subclavian artery. The Stanford classification has two types: type A dissections involve the ascending aorta, and type B dissections are those that do not involve the ascending aorta. Every dissection involving the ascending aorta (type A or I or II) has to be operated. Regarding the dissection involving the descending aorta (type B or III), the treatment is most often conservative around the world but many advanced centers have adopted endovascular treatment for cases with specific scenarios like untreatable pains, malperfusion, and pseudocoarctation with arterial hypertension in the upper body part [16–19].

## 4. Conclusion

Our case presents a unique scenario where a severe headache led to the patient's presentation to the emergency room that prompted further evaluation into his symptoms and led to the diagnosis of aortic dissection. The classical teaching professes that the classic chest pain syndrome radiating to the back should raise the suspicion regarding a dissecting aortic aneurysm. This case illustrates the importance of keeping in mind aortic dissection as a differential in patients with acute onset headaches in whom any intracranial source of headache is not found.
